# Correlation of lesion severity with bacterial changes in Treponeme-Associated Hoof Disease from free-roaming wild elk (*Cervus canadensis*)

**DOI:** 10.1186/s42523-024-00304-9

**Published:** 2024-04-22

**Authors:** Jennifer H. Wilson-Welder, Sushan Han, Darrell O. Bayles, David P. Alt, Carly Kanipe, Kyle Garrison, Kristin G. Mansfield, Steven C. Olsen

**Affiliations:** 1 Infectious Bacterial Diseases of Livestock Research Unit, National Animal Disease Center, Agricultural Research Service– USDA, 1920 Dayton Ave, 50010 Ames, IA USA; 2 Colorado State University Diagnostic Medicine Center, Fort Collins, CO USA; 3 Washington Department of Fish and Wildlife, Olympia, WA USA

**Keywords:** *Cervus canadensis*, Elk, Digital dermatitis, *Treponema*, Lameness, Treponeme-associated hoof disease, 16S sequencing, Metagenomics

## Abstract

**Background:**

Treponeme-Associated Hoof Disease (TAHD) is a polybacterial, multifactorial disease affecting free-ranging wild elk (*Cervus canadensis*) in the Pacific Northwest. Previous studies have indicated a bacterial etiology similar to digital dermatitis in livestock, including isolation of *Treponema* species from lesions. The lesions appear to progress rapidly from ulcerative areas in the interdigital space or along the coronary band to severe, ulcerative, necrotic, proliferative lesions under-running the hoof wall, perforating the sole, and contributing to hoof elongation, deformity, and overgrowth. Eventually the lesions undermine the laminal structure leading to sloughing of the hoof horn capsule. The objective of this study was to characterize the bacterial communities associated with hoof lesions, which were categorized into 5 stages or disease grade severities, with 0 being unaffected tissue and 4 being sloughed hoof capsule. We also wanted to determine if the etiology of TAHD through morphological changes was dominated by *Treponema*, as observed in hoof diseases in livestock.

**Results:**

The bacterial 16S rRNA gene was sequenced from 66 hoof skin biopsy samples representing 5 lesion grades from samples collected by Washington Department of Fish and Wildlife as part of a voluntary hunter program. Analysis of the relative abundance of bacterial sequences showed that lesions were dominated by members of the bacterial phyla Proteobacteria, Firmicutes, Spirochaetes, Bacteroidetes and Actinobacteria. In lesion samples, members of the genus *Treponema*, *Porphyromonas*, and *Mycoplasma* increased with lesion severity. Association analysis indicated frequent identification of *Treponema* with *Porphyromona*s, *Bacteroides* and other anaerobic Gram-positive cocci.

**Conclusions:**

The bacterial 16S rRNA gene sequencing confirmed the presence of *Treponema* species at all stages of TAHD lesions, treponeme specie-specific PCR and histopathology, indicating that the morphological changes are a continual progression of disease severity with similar bacterial communities. Association and abundance of these other pathogenic genera within lesions may mean synergistic role with Treponema in hoof disease pathogenesis. Characterizing bacteria involved in lesion development, and their persistence during disease progression, provides evidence for science-based management decisions in TAHD infected elk populations.

**Supplementary Information:**

The online version contains supplementary material available at 10.1186/s42523-024-00304-9.

## Background

**Fig. 1:**
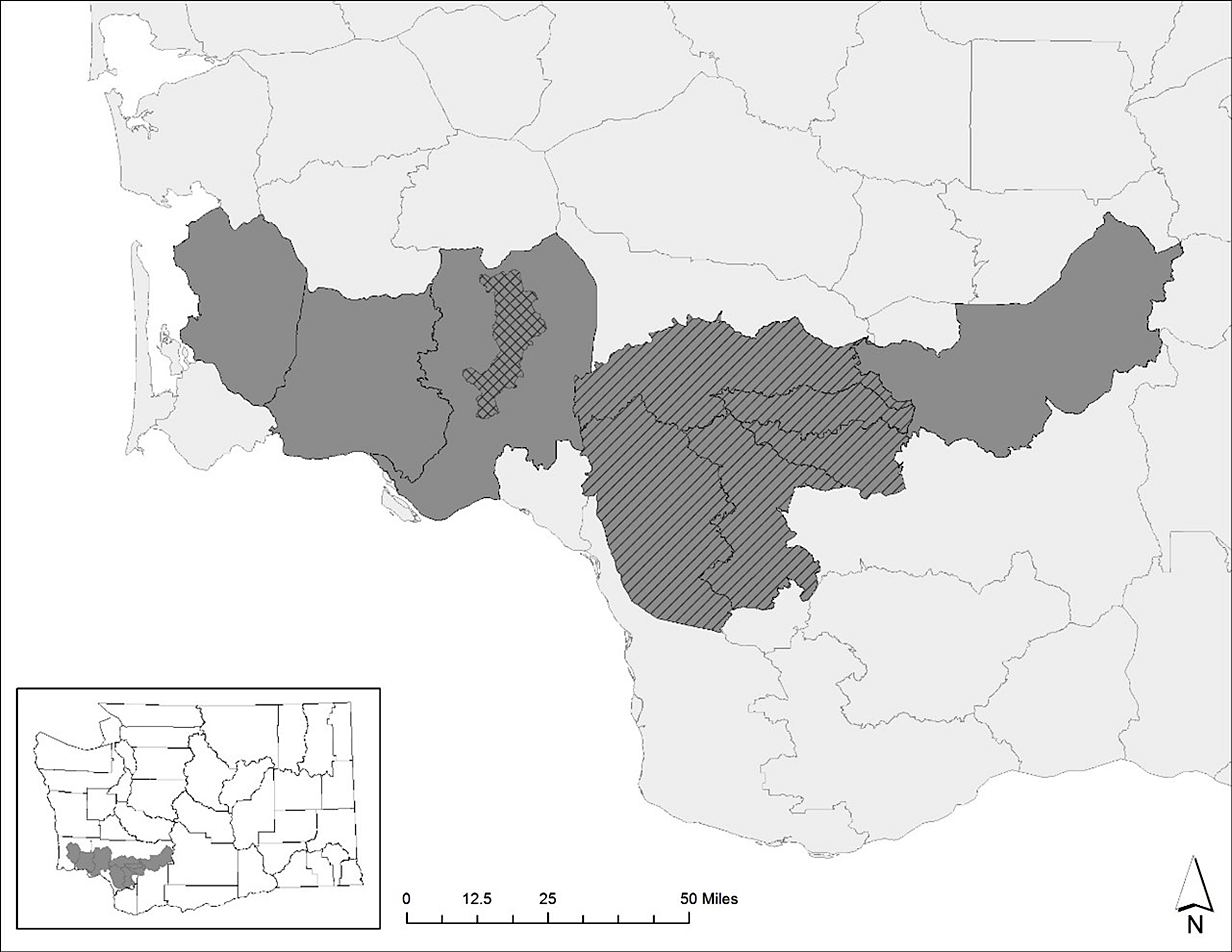
Map of Southwestern corner of Washinton State, United States, showing the regional game management units (GMUs). Shaded areas represent GMUs sampled in this study. Cross-hatched area is where TAHD was first documented in Southwestern Washington State, and the diagonally hatched areas represent the core GMUs of the Mount St. Helens elk herd, where the disease is highly endemic. Inset: Sampled GMUs overlaid on a map of counties in Washington State to illustrate geographical location of sampled GMUs in this study

Free-ranging elk (*Cervus canadensis*) in south-western Washington State were reported with deformed hooves and severe lameness. A series of investigations by Washington Department of Fish and Wildlife (WDWF) between 2009 and 2014 centered around the Mount Saint Helens elk herd (Fig. 1) determined that the hoof disease was most likely polymicrobial in origin and associated with infections of treponeme spirochetes within lesions [1]. No other underlying systemic disease was identified, although all elk in this region are known to have dietary deficiencies in selenium and copper [2]. Investigations determined that elk calves as young as seven months old showed hoof abnormalities and evidence of chronic infection [1]. *Treponema* isolated from hoof lesions had a high degree of genetic similarity to *Treponema* isolated from digital dermatitis lesions of livestock, leading to the condition being described as Treponeme-Associated Hoof Disease (TAHD) [1, 3].

### TAHD lesions

**Fig. 2:**
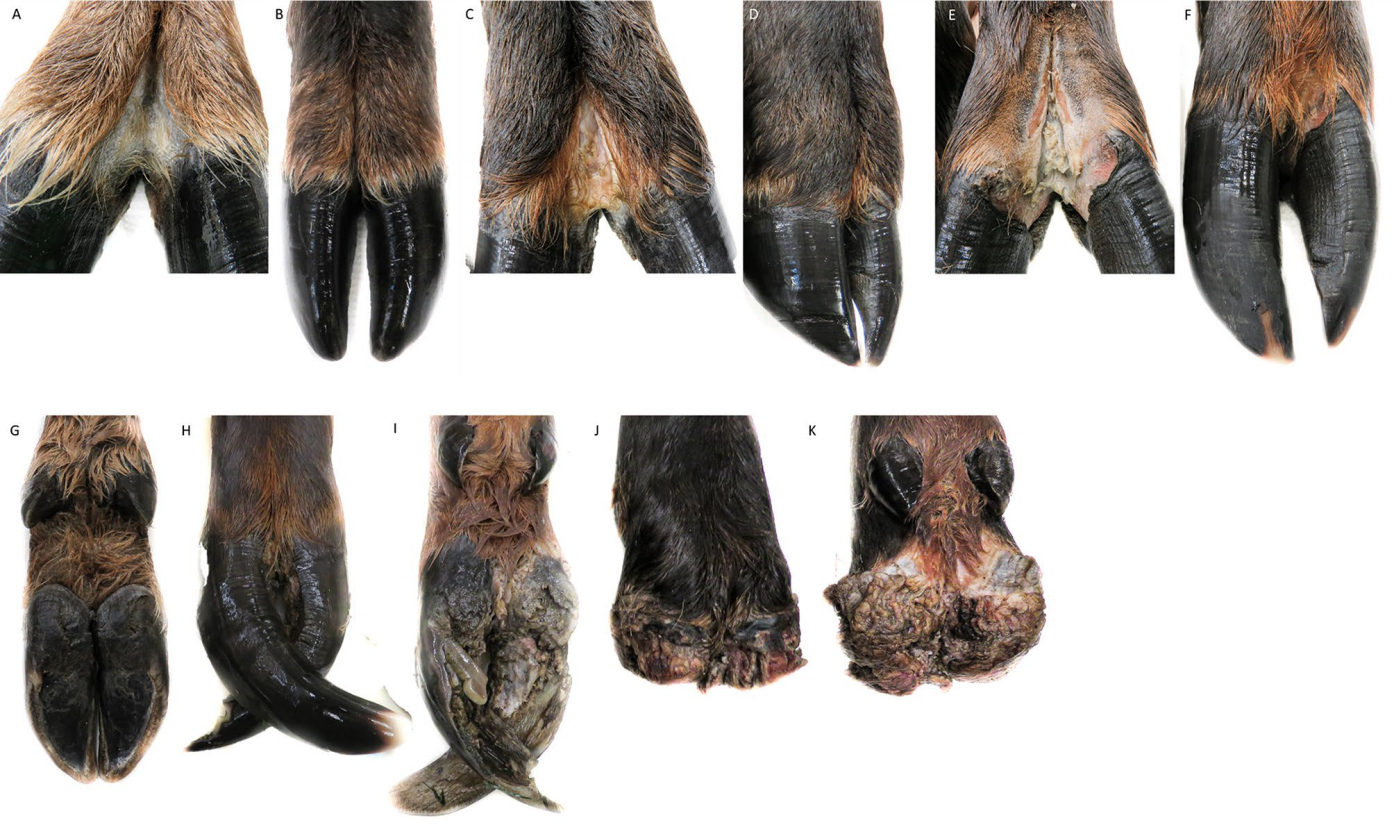
Lesion Grades. Photographs of representative grades of Treponeme-associated hoof disease (TAHD). **A** Interdigital space on foot with no lesions (No lesions, grade 0 Ng0): intact and normal looking coronary, interdigital skin, sole and heel. **B** Toe of normal length and wear pattern. No overgrowth or deformity evident. **C** Interdigital space of Grade 1 foot lesions (Yes lesions, grade 1, Yg1): Cutaneous lesions (ulcerative erosions) generally within the interdigital space but may be along coronary band or heel bulb. **D** Same hoof in C, showing no overgrowth or deformity. **E** Grade 2 foot lesions (Yes lesions, grade 2, Yg2): Ulceration evident in interdigital space, along coronary band, and medial undermining of the hoof capsule. **F** Hoof capsule overgrowth and deformity is evident. **G** Plantar surface of normal hoof with no lesions. **H** Grade 3 foot lesions (Yes lesions, grade 3, Yg3): Sole ulceration with inflammation/necrosis of the epidermal and dermal lamina, extensive of the interdigital space and coronary band. **I** Extensive hoof capsule overgrowth is present. **J** Dorsal and **K** Plantar aspect of Grade 4 foot lesions (Yes lesions, grade 4, Yg4): Sloughing of the hoof capsule, presence of tissue granulation, necrosis and areas of active inflammation or infection

TAHD has many similarities to treponeme hoof diseases of livestock, digital dermatitis (DD) in cattle and contagious ovine digital dermatitis (CODD). In preliminary investigations, parallels between livestock lesions and TAHD were observed, including histopathological changes to the dermal architecture and bacterial species isolated from lesions [1]. Digital dermatitis in cattle follows a series of morphological stages of development including predictable changes in gross appearance. The most common scoring system used in livestock is the M-score with M1 and M2 being active lesions, M3 healing lesions, M4 chronic lesions, M4.1 chronic lesions with active foci, and M5 normal skin [4]. As lesions move through gross or macroscopic stages, progressive histologic changes can be observed. Microscopically, DD is marked by epithelial ulcerations, parakeratotic hyperkeratosis, invasion of the stratum spinosum or papillary dermis by dense mats of spirochete bacterial forms and multifocal to diffuse suppurative infiltrates (neutrophils) [5]. It is not unusual for gross and histologic lesions to differ, i.e., visibly resolving (M3) or inactive (M4) lesions and yet histologically active [5–7]. Similarly, CODD demonstrates progression through several stages of severity and a similar 5-point grading scale has been proposed, with Grade 1 being erosion or ulceration at the dorsal coronary band to Grade 4 being complete loss of hoof-horn capsule and active lesion area but evidence of regrowth with Grade 5 being a healed foot. Histological changes are also observed in ovine lesions, including lymphoplasmacytic dermatitis with hyperplasia and orthokeratotic hyperkeratosis, epithelial erosions, suppurative inflammation, intracorneal pustules, and intralesional hemorrhage [8]. Descriptions within CODD lesion grades correlate with lesion observations in TAHD, as the syndrome in elk begins with ulcerative skin lesions and progresses to loss of the hoof-horn followed by degradation of the distal phalanx (Fig. 2). The CODD lesions scale was used to develop a lesion grading scheme for TAHD [1, 8].

In both DD and CODD, increased severity of lesions is associated with marked increases in *Treponema* numbers, diversity, and depth of invasion [9–13]. Three species of *Treponema*: *T. phagedenis, T. pedis* and *T. medium*, have been identified globally in association with DD, both geographically across the globe, associated with hoof lesions in multiple hosts (goats, sheep, cattle, elk, bison, Mediterranean buffalo), and persists in DD transmission animal models [13–18]. Other *Treponema* species that have been isolated from DD lesions less frequently or identified by bacterial 16S DNA sequencing include: *T. refringens, T. brennaborense, T. denticola* or *T. dentocola-*like, *T. lecithinolyticum, T. putidum*, and un-named less-characterized phylotypes (PT1, PT2, PT3, PT8, PT13) [14, 19–22]. Bacterial population differences between studies may reflect variations influenced by climate, geography, or husbandry practices. Recent meta-analysis of bovine digital dermatitis microbiota concluded that bacteria from the *Treponema* genus were highly abundant in bacterial populations in DD positive skin samples as compared to DD negative skin samples, implicating the genus as a core pathogen for lesion development [23]. There is evidence from numerous studies that DD and DD-like diseases (CODD & TAHD) are polymicrobial and that a core consortium of *Fusobacterium* spp., *Bacteroides* spp., *Porphyromonas* spp., and *Mycoplasma* spp., may also contribute to lesion development, although reported bacterial population abundances within lesions differ between published studies [1, 9, 11, 13, 17, 23, 24]. Molecular techniques have confirmed observations of spirochetal bacteria infiltrates deeper within the lesions, with cocci and fusiform rods forming matts on the outer epithelium in silver-stained tissue sections [1, 4, 25].

In DD lesions of cattle, bacterial populations change as lesions progress from active (M1 and M2) to chronic (M4) [4]. Some have suggested that overall bacterial diversity decreases, becoming dominated by *Treponema* as lesions become more chronic, whereas other reports indicate that overall diversity is maintained [9, 13]. While CODD lesions can develop *de novo*, some suggest it is initially a progressive infection initiated by *Fusobacterium* spp., *Dichelobacter nodosus*, and other opportunistic, pathogenic anaerobes similar to foot-rot, but ultimately, progress to lesions consistent with CODD with the invasion of *Treponema* [11].

### Study objective

**Fig. 3:**
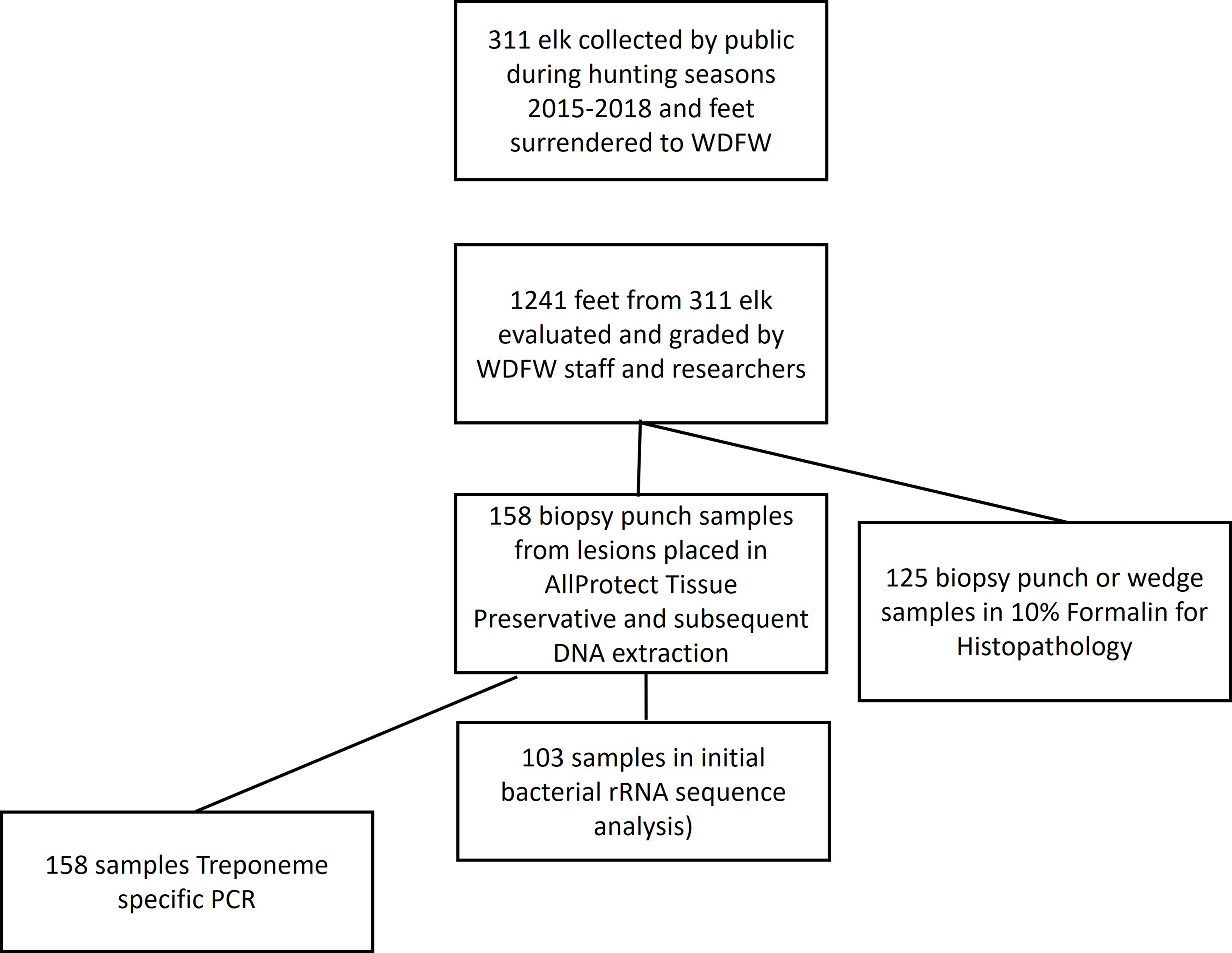
Flowchart of sample usage for subsequent analysis

The objective of this study was to characterize bacterial populations associated with elk hoof lesion grades and correlate population structure to lesion grade or gross observations. We hypothesized that lesion grades reflect disease progression, and that the composition of bacterial communities differ between grades and from populations in elk hooves without lesions. Elk hoof samples were opportunistically collected by the Washington Department of Fish and Wildlife (WDFW) between 2015 and 2018 in an area endemic for TAHD (Fig. 1). Normal feet, feet with TAHD lesions, and contralateral feet, were scored for lesions using visual (Fig. 2) and histological techniques, and bacterial communities characterized by molecular techniques. Sample collection and processing is summarized in Fig. 3.

### **Results**

#### Gross lesions

Results from scoring of feet are summarized in Table 1. Over the total study, samples from 311 individual elk were scored. Most hoof samples (77%) were determined to be normal, without gross lesions (lesion score 0). 10% were as scored as grade 1, 3% as grade 2, 3% as grade 3, and 5% as grade 4. Since the feet were collected in the same endemic area, we did not expect or observe differences in distribution of lesion scores between years in the study (Table 1). Approximately 73% of TAHD lesions were on hind limbs.

**Table 1:** Number of samples collected by year and lesion grade

Year collected	Number of elk	Number of feet^a^	Number of feet with lesion grade:					Number of animals with more than one diseased foot	Feet with lesion (no grade recorded)	Feet with no score or data recorded^b^	Number of feet with lesion (any grade)	Number of lesions (any grade) on hind feet	Percentage of lesions (any grade) on hind feet
			4	3	2	1	0						
2015	74	293	11	9	12	30	212	22	16	3	78	54	69
2016	69	276	16	11	7	25	209	14	0	8	59	45	76
2017	98	392	14	16	11	46	304	23	0	1	87	62	71
2018	70	280	17	4	7	26	226	16	0	0	54	42	78
Total number	311	1241	58	40	37	127	951	75	16	12	278	203	73
Total proportion (%)			5%	3%	3%	10%	77%	24%	1%	1%	22%		74

^a^Not all feet were scored

^b^Other animal information was recorded, no score was recorded

### Histopathology

Histopathology was used to confirm any potentially degraded samples identified by visual inspection. Presence of spirochetal forms and key markers of TAHD (inflammatory cells, acanthosis, and keratin crust thickening) as defined in previous studies was used to categorize lesions between grade 0 and grade 1 (Supplementary Material 3) [1, 17].

### PCR

Samples from 158 elk were screened by PCR for three *Treponema* species which have been previously found in TAHD and are associated with bovine DD and CODD lesions [3]. At least one of the three treponeme species was detected by standard PCR in 28% of samples from apparently healthy elk, i.e., Grade 0, no lesions on any feet. In contralateral samples from elk with lesions, the *Treponema* species were detected in 35% of samples. In general, the percentage of samples where *Treponema* was detected by PCR increased with lesion severity, as did the frequency of detecting two or all three species within a single sample (Table 2). *Fusobacterium* spp. detection based on presence of PCR amplification product was only assayed for in samples from the 2018 collection. From the 56 samples assayed, three contralateral samples (5%) and 14 lesion samples (14%) representing all four lesion grades, were positive for *Fusobacterium* (Supplementary Material 3). Interestingly, two of the positive contralateral samples were from animals that were PCR negative for *Fusobacterium* on the lesion sample.

**Table 2:** Result of PCR assay for three species *Treponema* associated with TAHD in samples collected 2016, 2017 and 2018

Lesion grade	Number of samples	Treponeme PCR(+)	Percentage of samples tested	*T. phagedenis*	*T. pedis*	*T. medium*
H (0)	32	9	28%	2	3	7
C (0)	49	17	35%	5	9	9
1	30	18	60%	9	14	7
2	12	8	67%	6	6	7
3	16	13	81%	7	12	9
4	19	18	95%	13	12	14
Total	158	83	53%	42	56	53

### Bacterial 16S rRNA sequencing

**Fig. 4:**
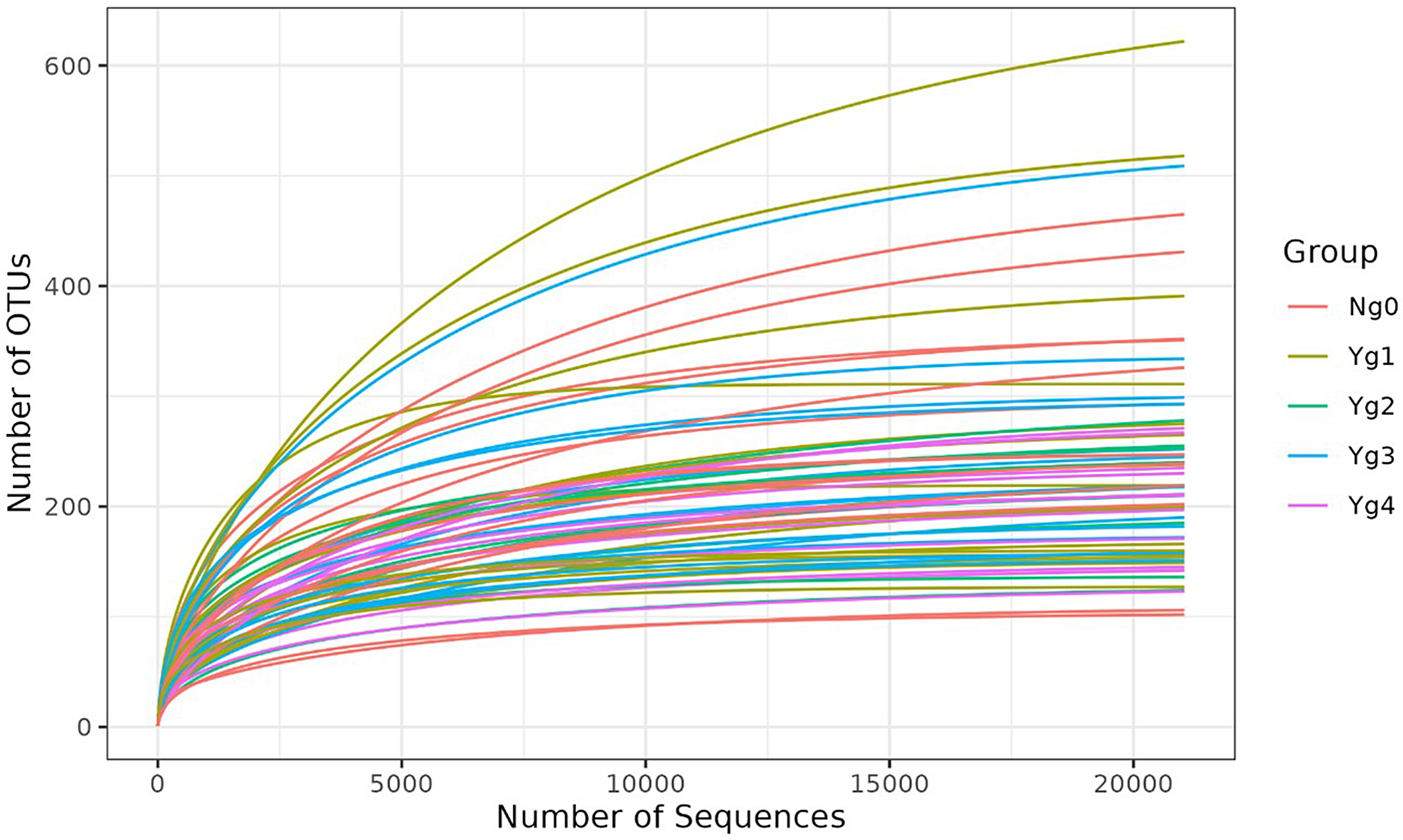
Rarefaction curve based on observed number of operational taxonomic units (OTUs) by number of sequences in each sample. Individual samples shown colored by lesion grade group

**Fig. 5:**
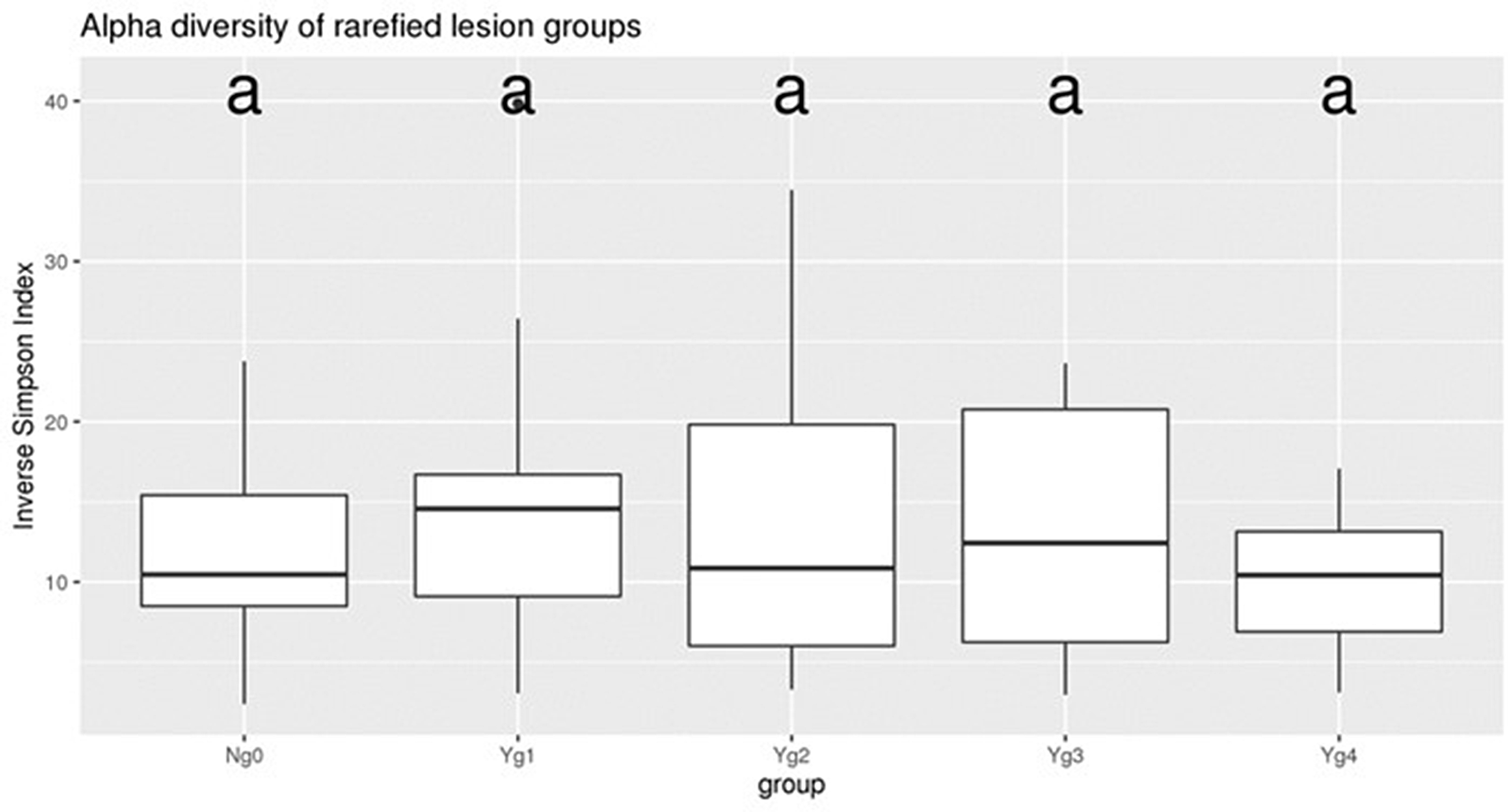
Inverse Simpson Index plot of Alpha diversity of lesion grades. Using rarefied data, and Tukey HSD, there was no significant difference between apparently healthy feet (Ng0) and the four lesion grades (Yg1, Yg2, Yg3, and Yg4)

**Fig. 6:**
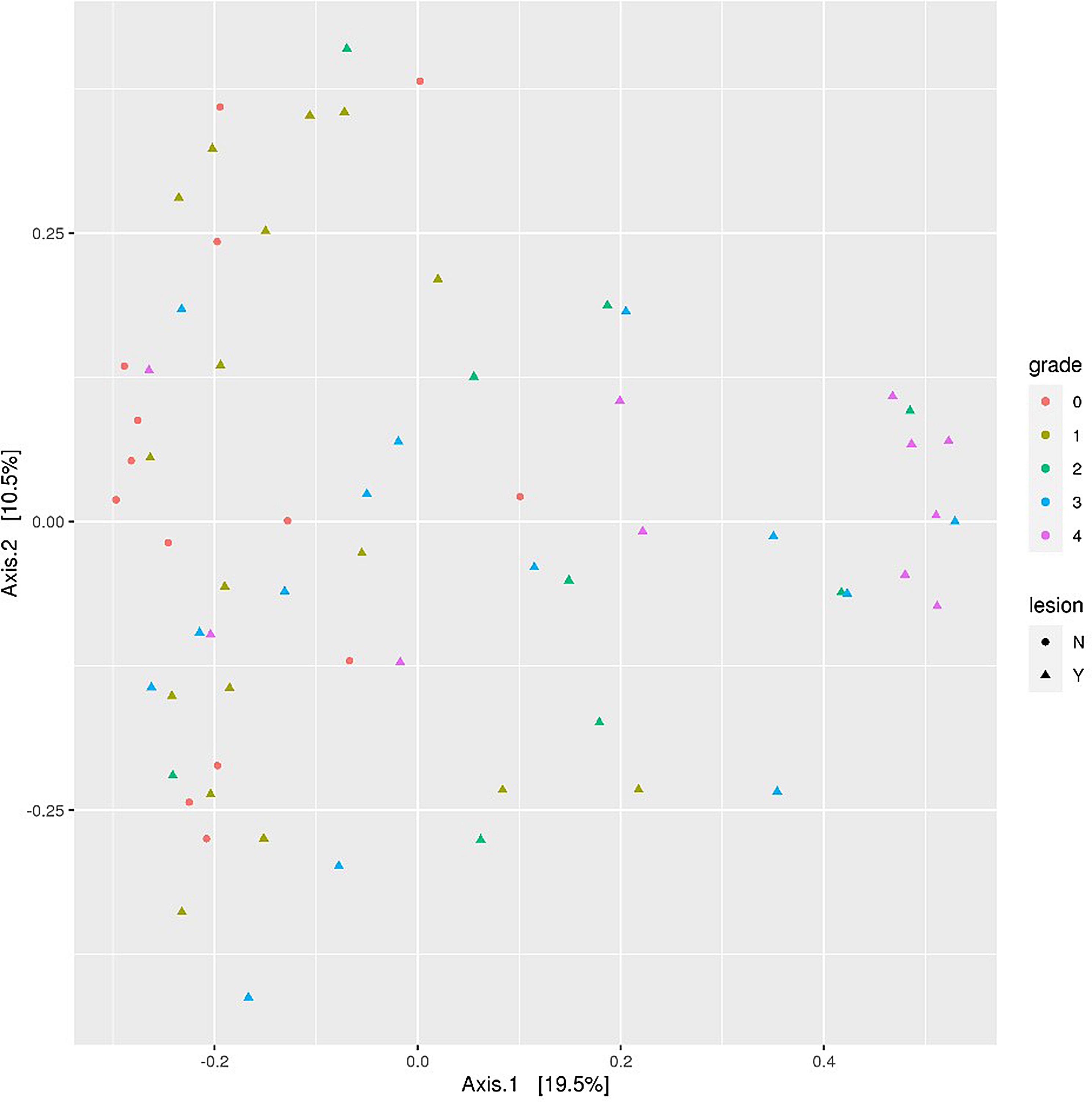
Beta diversity ordination plot using Bray-Curtis Principal Component Analysis

For 16S rRNA sequencing analysis, 68 total samples representing healthy feet and grades 1–4 were sequenced with an average 97,331 read pairs per sample (23,998 min, 135,131 max). Rarefaction curves were generated based on the observed number of OTUs as a function of sampling depth in each sample. The rarefaction curves approached OTU saturation the selected sampling depth of > 22,000 reads, thus providing reasonable estimates of microbial diversity (Fig. 4). Sampling depth was chosen based on the sample with the lowest number of sequences. The average number of operational taxonomic units (OTUs) per sample were 234 (min 84, max 661 OTUs). The DESeq2 analysis used the full (i.e., not subsampled) dataset. Data was analyzed by lesion (Y or N) and by lesion grade (g0, g1, g2, g3 or g4). Samples from the contralateral feet (35 samples) were excluded from analysis due to a lack of any discernable pattern. Alpha diversity was consistent across all grades sampled (Fig. 5) with no statistical difference (*P* > 0.05) between any of the lesion grade categories. Adding additional categories of grade (group) and location of the biopsy (intradermal vs. heel bulb, etc.) did not influence (*P* > 0.05) alpha diversity (Supplementary Data 5 Figure S1). Bray-Curtis principal component analysis was used to visualize taxonomic patterns based on relative abundance across samples (Fig. 6), with a diffuse clustering of the non-lesions (N0) on the left side of the graph and more of the severe lesions (Y, 1, 2, 3, 4) on the right side of the graph, but overall lacking tight clusters for each lesion grade.

**Fig. 7:**
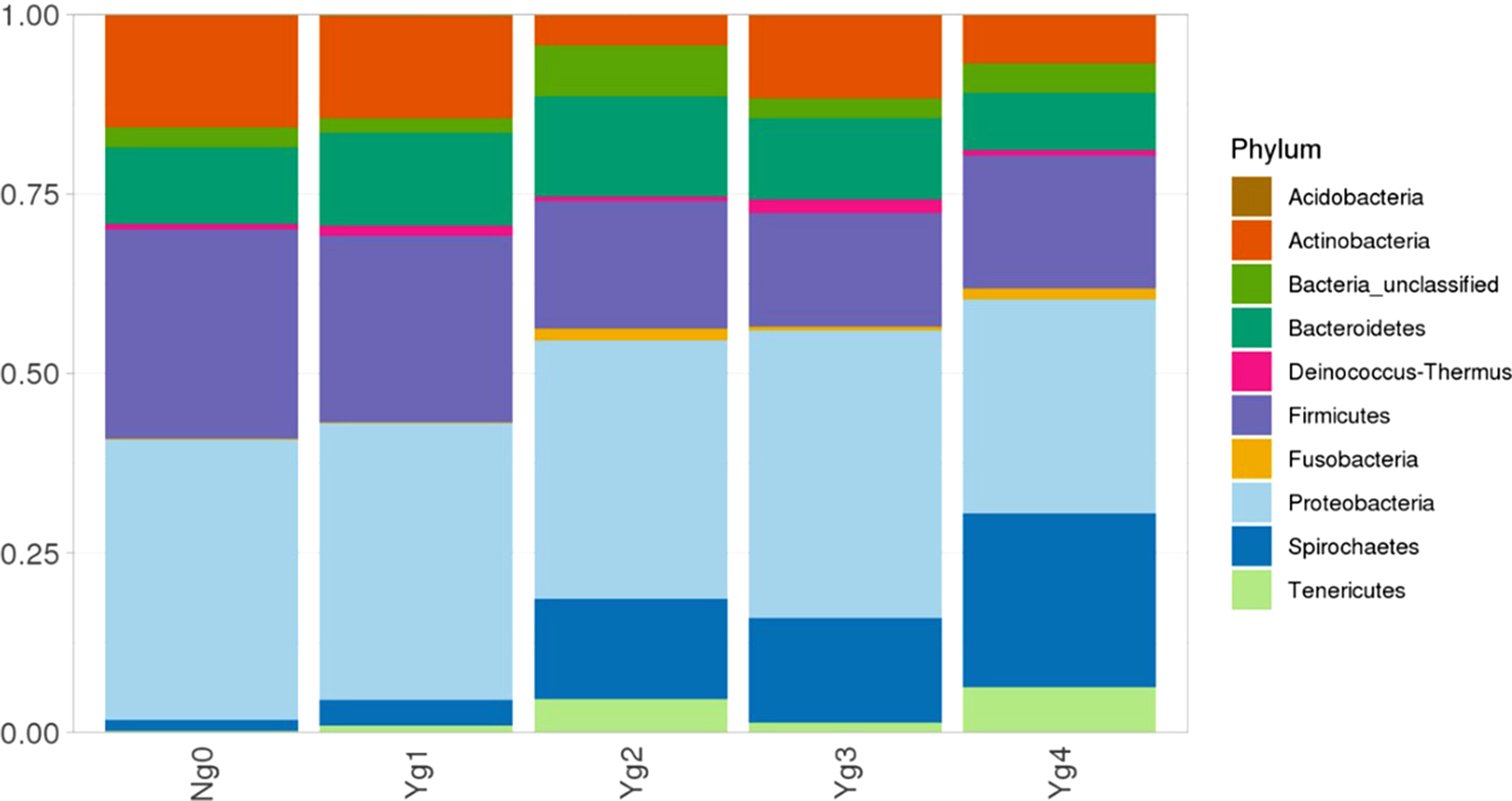
Top 10 bacterial Phyla by relative abundance in healthy/no lesion samples, lesions grades 1 to 4 (Yg1-Yg4)

**Fig. 8:**
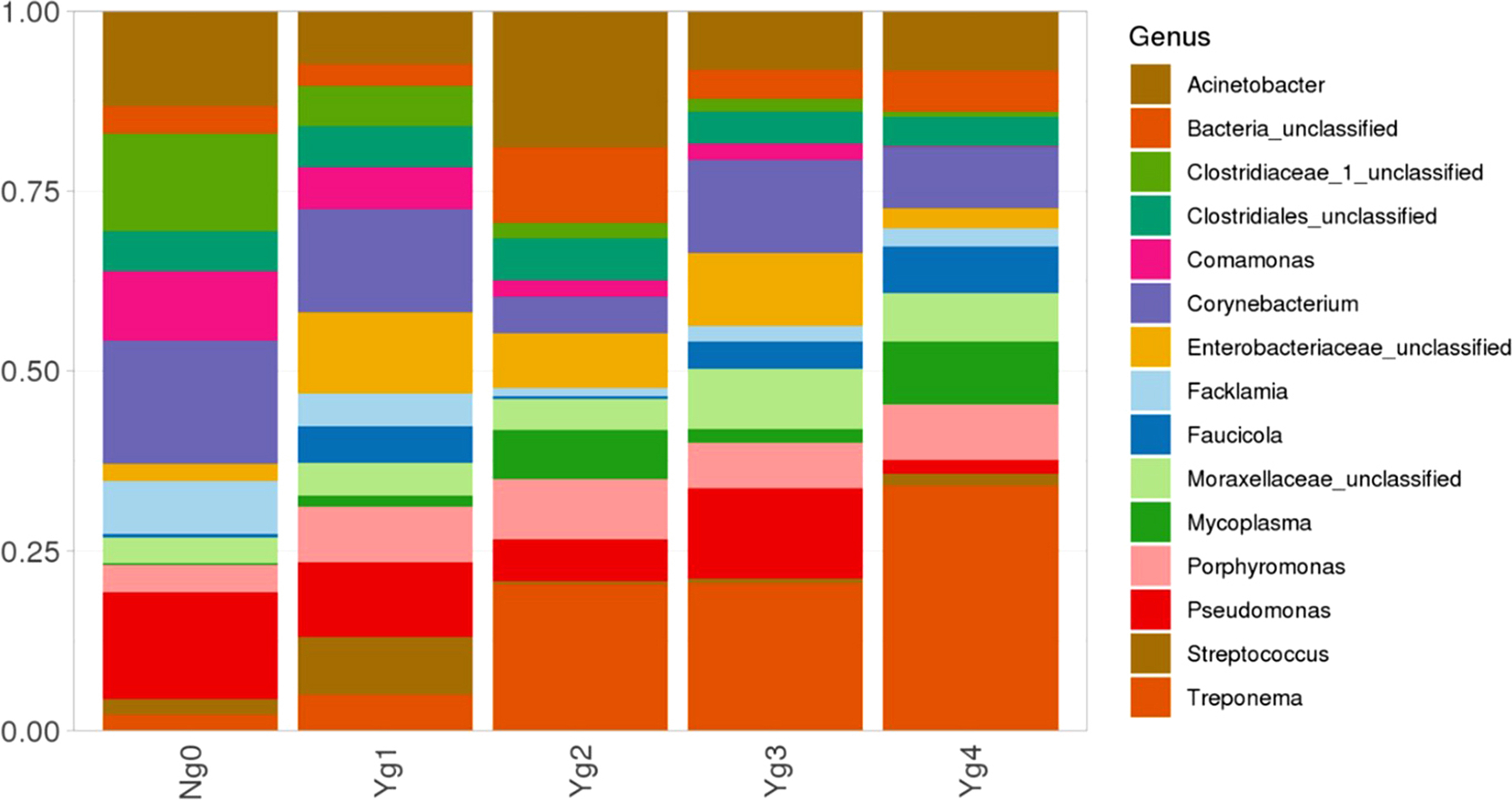
Top 15 bacterial Genus by relative abundance in healthy/no lesion samples, lesions grades 1 to 4 (Yg1-Yg4)

The top ten phyla for each lesion grade are depicted in Fig. 7, with all lesion grades dominated by Proteobacteria (30–40%), Firmicutes, and unclassified bacteria. While *Spirochaetes* are found in all lesion grades, including no lesion samples (Ng0), they represented increased proportions of relative abundance in more severe lesions, grades 2 (Yg2), 3 (Yg3) and 4 (Yg4) (Fig. 8). In a similar manner, genus *Tenericutes, Mycoplasma, Enterobacteriaceae, Fauciola*, *Porphyromonas* and *Fusobacteria* relative abundance was greater (*P <* 0.05) in grades 2–4 as compared to grade 1 or grade 0/no lesions. No lesion samples were dominated by genus *Acinetobacter*, unclassified *Clostridiaceae*, *Corynebacterium*, or *Pseudomonas*. Early lesions, Yg1, differed from both no lesions and grades 2–4 by having a higher relative proportion of *Streptococcus* species. By using differential heat tree matrix to visualize the differences in beta diversity across lesion scores (Supplementary Material 5 Figure S2), data suggests greater changes in type or genera and magnitude (relative abundance) between Ng0 or Yg1 and more severe lesions (Yg2, Yg3, or Yg4), with fewer differences in bacterial genera or magnitude between severe lesion scores when compared only among themselves.

When evaluating a core grouping of pathogenic organisms common to all lesion grades, 127 OTUs were significantly increased or decreased in pairwise comparisons between samples with no lesion (N) and other lesion grades (Supplementary Material 1). 55 OTUs were increased (*P* < 0.05) in 2 or more lesion grades: 3 OTUs in all 4 lesion grades, 20 in 3 of 4 lesion grades, and 31 in at least 2 lesion grades. Of those 55 OTUs, 9 were *Spirochaetes*, 15 were *Firmicutes*, 13 were *Bacteroidetes*, and remaining phyla represented *Actinobacteria, Proteobacteria, Deinococcus-Thermus, Tenericutes*, and SR_1. Only 20 OTUs were significant in three out of four lesion grades compared to no lesion, (Supplementary Material 5 Figure S3) including representing genus *Treponema*, unclassified Bacteria, *Mycoplasma*, *Bacteroidetes*, *Campylobacter*, *Clostridiaceae*, *Lachnospiraceae*, *Anaerococcus* and SR_1. Three OTUs were significantly different in all 4 lesion grades compared to no lesions (Supplementary Material 5 Figure S4) consisting of an OTU matching genus *Mycoplasma* and two matching *Bacteroidetes*. There is considerable variance across all of the significantly abundant OTUs in the copy number per samples and relative slope of trendline drawn across lesion grades between OTUs and bacterial families (Supplementary Material 5 Figures S3 and S4) as was predicted in the Beta diversity analysis (Fig. 6).

**Fig. 9:**
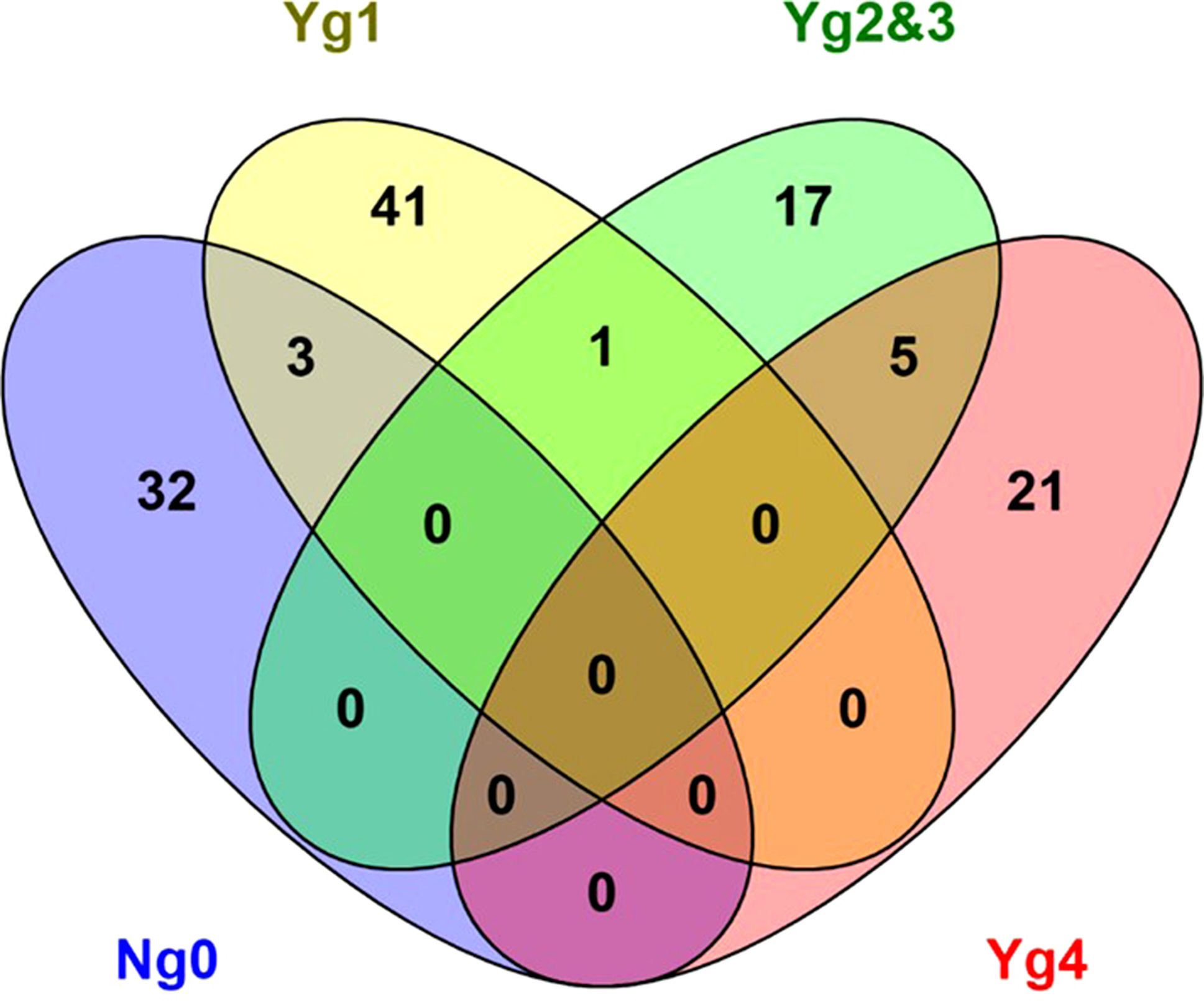
Venn diagram of distribution of association rules across lesion grades given the constraints of Lift greater than 5 and present in greater than 20% of the samples in each lesion category. Only one association was given for Yg3 which overlapped with Yg2, thus the two categories were combined

To further examine interactions between bacteria and lesion grade, data was analyzed using unguided rule association generator analysis. Output was further limited to rules with a lift > = 5 to better identify actionable rules and adding another filtering condition that more than 20% of the samples had to contain the rule same rule, rule association analysis returned 120 rules or pairings of OTUs that were associated with each other. This limited the single associations and other rare or unique combinations. The distribution and overlap of common association rules are illustrated in Fig. 9 and listed in Supplementary Material 4. Five associations were common between grades 2/3 and grade 4, only 1 association was common between grade and grade 2/3, and 3 associations overlapping between 1 and 0 with no overlapping associations between grades 1 and 4, or 0 and 2/3, or 0 and 4. Each grade had many unique associations of high probability (Lift > 5) but did not meeting the 20% sample threshold.

For lesion grades 2 and 4, *Trepomema* were most frequently associated with other *Treponema* OTUs (8 rules), followed by order *Bacteroidales* (5 rules), including genus *Porphyromonas*. Treponema was also associated with *Tissierella* and *Desulfovibrio* (2 rules). Other interbacterial associations revealed in the association analysis was with a group of Family *Clostridiales*, generally known as Gram + anaerobic cocci (GPAC). These GPAC include genus *Anaerococcus*, *Peptoniphilus, Ruminococcaceae, Peptoniphiliaceae, Lachnospiraceae* were found in frequent associations with *Treponema, Bacteroidales*, and *Porphymonas*.

## Discussion

Wildlife disease researchers operate at a disadvantage compared to livestock disease researchers as many types of population-based data require large sample sizes to make statistically significant inferences; relatively easy with livestock but to difficult obtain from wildlife under natural conditions. Sampling during live capture and with release afterwards is expensive and can cause excessive stress or injury to the animal. Terminal collection of sufficient numbers of animals for scientific study, even if the species is plentiful, is generally not publicly acceptable, especially with a highly desirable hunted species like elk. Therefore, using “samples of opportunity” and coupling scientific inquiry with other management activities becomes a viable avenue for data collection while conserving animal resources. For TAHD samples, collection was coordinated with WDFW samples from elk that were legally hunted in the region of interest (Fig. 1). For this reason, our data should not be considered a survey of disease prevalence in the elk population. In the current study, 311 animals were evaluated, an average of 70 per year, but the estimated elk harvest (reported legal hunts) of these game management units range from 641 in 2015 to 375 in 2018 (Data source: Kyle Garrison, WDFW). A high proportion of normal or healthy feet was expected in the collected samples as it was assumed that hunters would select healthy animals for harvesting. Prevalence index for TAHD in the sampled area suggests that approximately one-third of the greater Mount Saint Helens elk population is affected by TAHD (Data source: Kyle Garrison, WDFW). However, 77% of our hunter collected samples had no visible lesions. Grading feet for lesions was especially difficult using previously frozen and thawed limbs, especially for early grade 1 lesions, as serous exudate and erythema usually present in live or recently deceased tissue was absent. It was of interest that 76% of the lesions, regardless of grade, were on hind limbs (Table 1). Both DD and CODD have shown increased frequency of lesions on hind limbs [26, 27]. In cattle, it has been hypothesized that predilection for DD lesions on hind limbs was due to increased time of exposure to manure and contagious material, especially in high-production housing conditions. However, the similar observation in free-ranging elk suggests other factors may contribute to disease development. One hypothesis is that mechanical or physical strain on the hind feet predilect the skin to micro-abrasions allowing for bacterial invasion. A second hypothesis is that the hind feet are often under the animal while ruminating creating an incubated environment compared to the front hooves. Regardless, the trend for hind limbs to be affected in bovine DD, CODD and TAHD indicates that while high production housing and manure slurry may contribute or be a risk factor for digital dermatitis, the actual causes behind this hoof disease are varied and complex.

In general, PCR detection of the three *Treponema* species (*T. phagedenis, T. pedis* and *T. medium*) correlated with severity of lesions. The likelihood of detecting more than one of these three treponemes (*T. phagedenis*, *T. pedis* and *T. medium*) increased with lesion severity. Some have questioned the usefulness of PCR detection given reports of numerous false negatives [28]; however, it is worth noting those studies used surface swabs, which may not be sufficient to detect *Treponema* deeper in the lesion. Treponemes are more commonly seen at the leading edge of active lesions and within the tissue layers. This discordance between surface swab and biopsy results has also been observed in CODD [11]. By using biopsy tissue, we were able to achieve good correlation between visual gross score and PCR detection for one or more tested *Treponema* species. Other potential problems with PCR based assays include false positives due to the enhanced sensitivity of nested PCR assays. It should be noted we detected numerous positive *Treponema* PCR responses in contralateral samples (Table 2). The histology on many of these contralateral samples (Supplementary Material 3) suggest the sample was normal or healthy tissue. In these cases, PCR and 16s rRNA sequencing was possibly picking *Treponema* or treponemal DNA from surface contamination from environmental sources, other hooves with lesions, or possibly during handling for sample collection.

We did not detect treponemes by PCR as frequently in late-stage grade 4 lesions where the coronary band and underlying lamellae were often completely compromised, and the hoof-horn or hoof capsule is gone (Fig. 2). Grade 4 encompasses a variety of presentations ranging from a freshly sluffed hoof (possibly during or just prior to when animal was harvested) with active areas of disease in the interdigital space to loss of the entire phalanx from above the dewclaw (septic and chronic condition). The joints are often swollen, and the interdigital space filled with granular and fibrotic tissue. With these gross lesions the region where *Treponema* and the original disease microbiota reside may be absent [1].

While there was an observed shift towards a greater relative abundance of spirochetes or *Treponema* in advanced stages of lesions was expected, the dominance of treponemes in lesion tissue was not as marked as observed in bovine digital dermatitis, where *Treponema* can account for 80% or more of observed OTUs [13, 23, 29]. The persistence of soil associated organisms with each of the lesion grades (*Deinococcus*, *Actinobacter* and many of the *Firmicutes*) was also not surprising and different from bovine DD. While the hooves were washed lightly during examination before taking samples, soil and contaminating organisms probably persisted on the skin. As the hooves become overgrown and mis-shaped (Fig. 3), they fill with soil and debris. This could also explain the high number of soil organisms that are associated with TAHD. There is a growing recognition that previously considered “normal skin microflora” may be opportunistic and pathogenic in damaged skin. Other drivers of known hoof infections, *Fusobacter* spp. and *Dichelobacter nodosus*, are not dominant genera but do demonstrate differences between lesion grades in the heat tree branches, especially when comparing grades 2 or 3 to no lesions or grade 4 lesions. These two organisms have been found sporadically in TAHD samples using species-specific PCR both previously and within this study (Supplementary Material 3) [1]. We observed an increase of *Mycoplasma* associated OTUs with lesion scores of 2 and 4, but not in hooves of lesion grade 3. Grade 3 lesions are lesions where the hoof horn wall is under-run, and the sole or pad of the hoof capsule is ulcerated (Fig. 3). The most bacterially active region therefore may be either under the hoof horn wall or in the hoof sole, and the bacteria in question may no longer be present in the interdigital space where most biopsies were taken in this study.

**Fig. 10:**
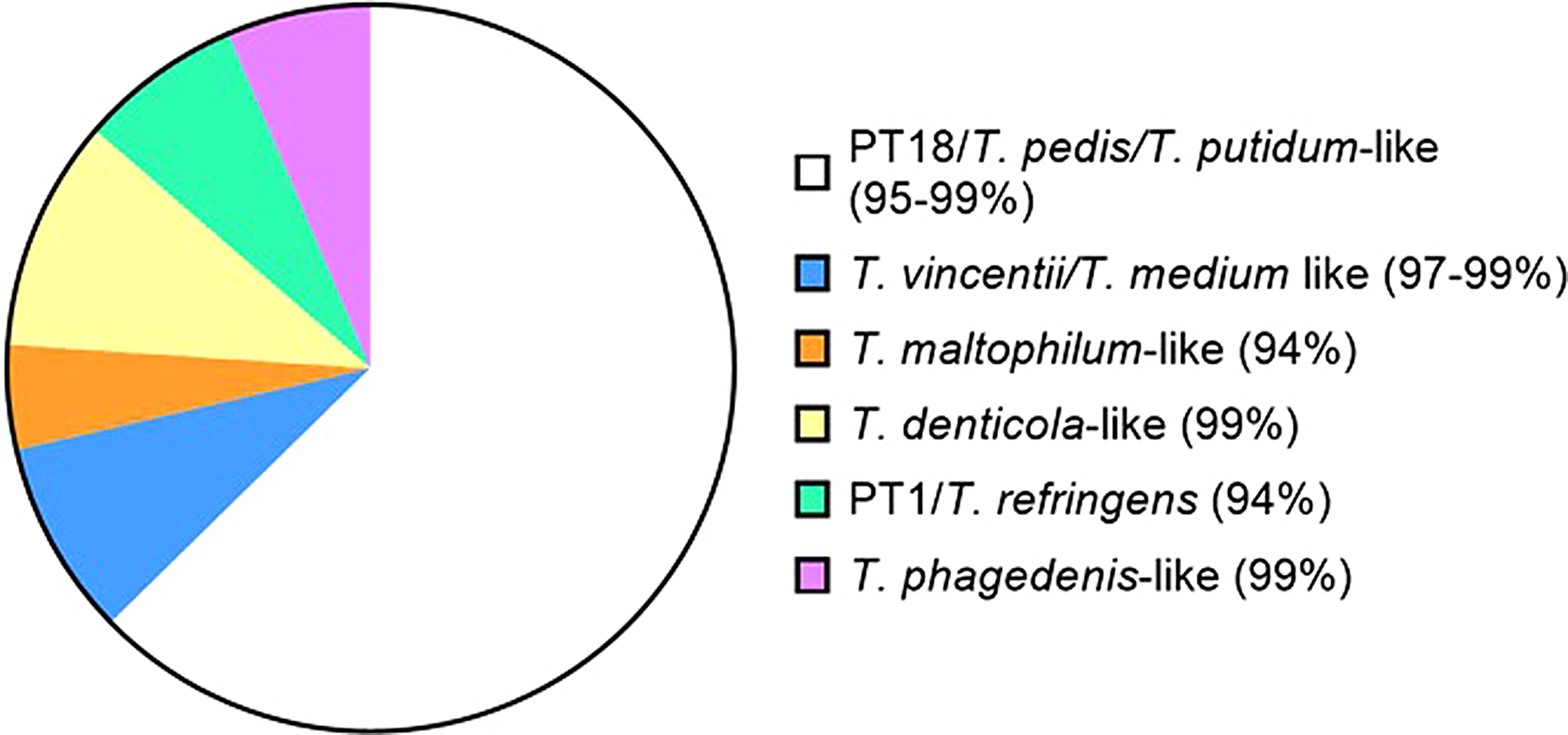
Pie chart depicting proportion of total OTU counts across all sequenced samples for the 14 OTUs associated with the 6 genogroups (by NCBI BLAST sequence alignment) (percentage of alignment match)

Caution must be taken when assigning species using 16 S rRNA sequencing, especially as many may not be distinguishable from analyses using the V4 region. With this caveat, we used the NCBI nucleotide BLAST function, to perform an alignment search of 5 randomly chosen V4 sequences within each of the 14 OTUs associated with *Treponema* (Supplementary Material 2). Each gave a high probably match (> 94%) to species in the NCBI database. 6 OTUs share high homology with *T. pedis*/PT18, 3 OTUs shared homology with PT9/*T. vincentii/T. medium*, 2 shared homologies with *T. maltophilum* and 1 OTU each shared homology with *T. denticola*, PT1/*T. refringes*, and *T. phagedenis* respectively. *T. vincentii* and *T. medium* are indistinguishable using the V4 region of 16 S alone [12, 30, 31]. Proportion of these 14 OTUs across all sequenced samples is graphically depicted in Fig. 10. While *T. pedis* and *T. medium* were expected based on established links for digital dermatitis of livestock and PCR data (Table 2), the low number of *T. phagedenis* associated OTUs was a bit unexpected. *T. pedis* seems to be the dominant treponeme in TAHD whereas *T. phagedenis* tends to dominate all stages of bovine DD [9, 13]. To date, *T. phagedenis* and an isolate in the *T. vincentii*/*T. medium*-like group are among the only cultured *Treponema* isolates from TAHD [1, 3].

Association rules, sometimes referred to as affinity analysis or market basket analysis, is an unsupervised machine learning approach for describing relationships between items and sets of items. In this study, we used association rules to identify relationships between individual OTUs and the groups of OTUs found in samples. Using this approach, we were able to detect OTUs that are found together more commonly than would be expected based on the occurrence of all OTUs in the samples. The identified associations do not show causality but instead provide information on key associations that might otherwise go unnoticed. While many of the bacterial association rules were to unclassified bacteria or with OTUs recognized only at the phyla level, some of the association rule data was as predicted by digital dermatitis disease of livestock. This includes OTUs identified from the order Bacteroidales, genus *Porphyromonas*, *Tisserella* and *Desulfovibrio* (Supplementary Material 4). These pathogenic bacteria have identified previously in digital dermatitis of livestock or in a similar disease, periodontal infections of humans and companion animals. Periodontal infections are polymicrobial with multispecies interactions potentially altering the immediate environment making favorable conditions for *Treponema* to invade [32]. Further linkage among the treponemes was observed in the rule association pattern discovery. Presence of OTU406 resulted in lift of 6.38 in association with OTU339 and the rule was present in both lesion grade 2 and lesion grade 4. Interestingly, OTU406 had alignment to the *T. pedis* group and OTU339 to the *T. vincentii/T. medium*-like group. Additionally, the association analysis revealed several associations between *Treponema* and members within the Family Closridiales, specifical the GPAC. GPAC include *Anaerococcus*, *Peptoniphilus, Ruminococcaceae, Peptoniphiliaceae, Lachnospiraceae*, among others depending on the system studies. They make up 25–30% of anaerobic bacteria isolated from clinical infections including polymicrobial wound infections, and are ubiquitous in human and animal gastrointestinal flora, and soil environments [33]. Eight different rules were between OTUs from Phyla Firmicute and Bacteroides, most of which are GPAC, and 11 rules were between 2 or more Firmicutes OTUs, further highlighting this interaction of GPACs in tissue infection. Because speciation cannot be accurately determined by 16 S rRNA sequencing if there is no reference in the database used, many OTUs are for “unclassified” within the order, family, or genus. It is not known if these *Bacteroides* and GPAC are indeed opportunistic residents of the skin, from the gastrointestinal tract of elk, or environmental residents (soil or plants). Regardless, the 7 association rules of *Porphyromonas*, *Ruminococcaceae*, and/or *Peptoniphilaceae*, and *Erysipelotrichaceae* suggest a pattern and possible pathogenic role for GPAC in hoof disease with *Treponema* [34, 35]. Many of the other unique rules within each lesion grade were associated with cyano-purple bacteria, and other bacteria commonly associated with soils, wet environments, and anaerobic marshy wetlands. Another surprise association was one that was lacking. Despite being significant in the DESeq2 analysis and having high relative abundance, *Mycoplasma* OTUs did not show up with enough regularity to meet the parameters (high Lift and > 20% of samples in a category) of the association rules. Thus, applying different types of analysis to the 16 S bacterial sequence data we have different evaluations of the abundance, comparison between sample grades and associations of bacteria (or bacterial types) within and between the sample grades to give a more complete understanding of the microbiome.

## Conclusions

The objective of this study was to characterize the microbiota of the 5 defined lesion grades of TAHD and determine if they represent a continuous disease process. Based on bacterial 16 S rRNA sequence comparisons of the microbiota of the represented lesion grades, we conclude that TAHD is a continuous process. As lesion severity increases there are increases in the relative abundance of the *Treponema*, and previously known pathogenic genera associated with digital dermatitis of livestock and polymicrobial skin infections. These *Treponem*a species align with the three dominant types previously identified in TAHD and other hoof diseases of livestock. While many recovered organisms were associated with soil and marsh environments, there was a strong association between *Treponema* and members of other opportunistic pathogenic groups and bacteria associated with lesion development. These pathogenic bacteria included increasing abundance of Bacteroides, *Porphyromonas*, *Mycoplasma* and GPAC. Association analysis highlighted the frequent correlation between *Treponema* and these pathogenic genera, especially the GPAC. Further study and analysis are needed to further characterize bacterial populations, if there is a synergistic relationship with *Treponema* or what the role is for these other bacteria in hoof disease pathogenesis for both elk and livestock.

## Materials and methods

Hoof samples were opportunistically collected over 4 years (2015–2018) by Washington Department of Fish and Wildlife (WDFW). Front and hind feet were voluntarily obtained surrendered from licensed hunters in specific game management units (GMUs) within Southwestern Washington state (Fig. 1). The area chosen for the monitoring program was located within the endemic disease area and associated with the Mount Saint Helens elk herd (Fig. 1). Hunters who harvested an elk in these areas the previous season were sent a letter explaining the project, hoof collection procedures, plastic collection bags and directions, data sheet for recording location, and a request to submit hooves from any harvested elk. Collection sites were located near hunting areas and submitted hooves were picked up frequently by WDFW biologists. Collection mostly took place in November, when ambient temperatures were relatively cool, helping to preserve the samples. Intact feet were stored whole, wrapped in plastic, at -20 °C until processing.

Experts from WDFW, Colorado State University Diagnostic Medicine and USDA Agricultural Research Service assisted with case diagnosis and sample collection. Between 6 and 12 months after collection, hooves were thawed at 4 °C for 24–48 h and rinsed with tap water. Scientists scored gross lesions with lesion grades ranging from 0 (healthy tissue) to 4 (sloughed hoof horn on one or both claws) as previously described (Fig. 2) [1]. Samples deemed to be well preserved and not degraded were used in the study. Hoof samples were collected from available material with a goal of obtaining bacterial population data from a minimum of 10 samples in each lesion score category and from corresponding contralateral or healthy feet, and from animals with no gross lesions on any feet (Grade 0). Approximately 24% of animals had more than one affected limb. Therefore ‘contralateral’ samples were obtained from feet with no lesions on the same diseased animals, not always the anatomically contralateral. Duplicate 5 mm biopsy punches or 1 cm^3^ wedges were used to collect tissue biopsy samples from the interdigital area or lesioned regions. One sample for histological evaluation, if required, was placed in 10% neutral buffered formalin and a duplicate biopsy sample was placed in 1 ml Qiagen AllProtect Tissue Reagent (Qiagen, Germantown, MD, USA) for DNA extraction and molecular analysis. Biopsy punches in Allprotect were transported at room temperature and stored at -80 °C on arrival until processed. Data does not reflect paired samples for all assays and all samples collected.

### Histopathology

Skin biopsy sections (*n* = 125) collected from active lesion material, generally at the interdigital space, or from contralateral hooves, were fixed in 10% neutral buffered formalin for a minimum of 48 h and then decalcified in a solution consisting of 5% formic acid and 37% formalin (Cancer Diagnostics, Durham, NC) for 24–48 h until pliable and easily sectioned with a scalpel [36]. Sections were then de-keratinized by immersing in a solution of 17% potassium hydroxide and 10% formalin (StatLab, McKinney, TX) for 16–48 h. Processed sections were paraffin embedded, trimmed to 7–10 μm, and stained with hematoxylin and eosin (HE) for microscopic examination. Replicate sections were Gram-stained with method of Brown and Ben as described previously or Steiner’s silver-stain for examining spirochete morphology [37, 38]. All prepared slides were examined by light microscopy by specialized pathologists.

### DNA extraction

Skin biopsy punches (*n* = 158, including healthy, lesion and contralateral samples), preserved in 1 mL Qiagen AllProtect (Qiagen, Germantown, MD, USA), were thawed on ice, removed from cryovials and blotted to remove excess liquid. DNA was extracted using Qiagen DNeasy Blood and Tissue kit following manufacturer instructions for tissue with overnight digestion. Total DNA yield was quantified (Qubit fluorometer, Life Technologies, Grand Island, NY) and DNA stored at -80 °C prior to downstream processing. The same DNA extracted sample was used for both Treponeme specific PCR and 16S rRNA sequencing.

### Treponeme specific PCR

PCR for *Treponema medium, Treponema pedis, Treponema phagedenis, Treponema vincentii*, *Treponema denticola, Treponema brennaborense* and *Fusobacterium necrophorum* was performed on *n* = 158, including healthy, lesion and contralateral samples, in a two-stage nested PCR assay as previously described [1, 17, 18]. Positive controls were genomic DNA purified from each respective bacterial species. Details on bacterial strains, PCR primers and amplification conditions are given in supplementary material 5 (Table S1). Sterile water was run in each PCR assay as a negative control. PCR reaction products were visualized by electrophoresis on 1.5% agarose gel stained with SYBR Safe DNA gel stain (Invitrogen Life Technologies, Carlsbad CA, USA).

###  S rRNA sequencing and analysis

Amplicon library preparation for bacterial 16S rRNA gene v4 region sequencing (*n* = 103 samples) was conducted as described (https://www.anl.gov/bio/environmental-sample-preparation-and-sequencing-facility). The sequencing done was 2 × 250 Illumina MiSeq v2 chemistry using universal 16S rRNA gene forward primers (515 F) and unique Golay barcoded reverse primers (806R) as previously described [13]. The V4 region is approximately 253 bp.

Reads for each individual sample were demultiplexed and binned according to barcode by idemp (https://github.com/yhwu/idemp). Overlapping R1 and R2 reads to form contigs result in near full-length coverage of V4 region, 243 to 254 bp in length. Reads were checked for quality and processed into operational taxonomic units (OTUs) using mothur analysis software (v.1.43.0) following the strategy outlined by Kozich et al. and https://mothur.org/wiki/miseq_sop/ (accessed January 2021) [39, 40] (i.e., following Lab MiSeq SOPs). The 16S rRNA gene v4 sequences were aligned to the Silva bacteria reference database (release 132) and taxonomy was assigned based on the mothur-formatted version of the RDPtraining set (trainset16_022016). Sequences were clustered into OTUs based on the pairwise distances between the unique sequences. Further data analyzes and statistical comparisons were made using R and the following R-packages “DESeq2”, “metacoder”, “microbial”, “OTUbase”, “phyloseq”, “taxa”, and “vegan.” The analyses also employed the following general R-packages: “dplyr”, “ggplot2”, “gplots”, “magrittr”, “RColorBrewer”, “reshape”, “scales”, “tidyverse”, and “VennDiagram” (https://CRAN.R-project.org/package=microbial) (https://CRAN.R-project.org/package=vegan) [39, 41–44]. The data was rarefied before comparative analyses except for the DESeq2 analysis that was performed with unrarefied data. Sequences were rarified to 21,040 sequences using the vegan package rarecurve function. This was the number of curated sequences in the smallest group, and based on rarefication curves, adequately captured diversity in the samples, thus no samples were dropped by rarefication. The inverse Simpson index was calculated using vegan package diversity function applied to the rarefied data. The rarefied data was converted to a phyloseq object, and relative abundance histograms were created using the microbial package plotbar function.

Pattern discovery was explored using Association rules as implemented in the arules v. 1.7-6 R-package [45]. OTUs with five or more reads within a sample from the Mothur analysis were listed in a csv file. The OTUs per sample data was read into a sparse matrix via the arules read.transactions() function. The apriori() function was implemented with the following parameters (support = 0.07246, confidence = 0.5, minlen = 2, maxlen = 4, and maxtime = 20). The resulting rules were filtered to remove rules with exactly the same items (OTUs). Finally, we evaluated the antecedent and consequent information for rules where lift was initially set at > 3. Data output is contained in Supplementary Material 4.

### Electronic supplementary material

Below is the link to the electronic supplementary material.

Supplementary Material 1

Supplementary Material 2

Supplementary Material 3

Supplementary Material 4

Supplementary Material 5

## Data Availability

Raw data collected from hoof examination, PCR, gross and histologic observation, and location data are included in Supplementary Data Files_metadata. 16S rRNA gene sequencing data (fastq files) can be accessed under NCBI Bioproject accession number PRJNA1070655 (https://www.ncbi.nlm.nih.gov/bioproject).

## Declarations

Work was completed by U.S. Department of Agriculture employees in the course of their assigned duties in relation to project number 5030-32000-223-00-D. Mention of trade names or commercial products in this study is solely for providing specific information and does not imply recommendation or endorsement by the U.S. Department of Agriculture. USDA is an equal opportunity provider and employer.
