# Supplementary material for: Correlation of lesion severity with bacterial changes in Treponeme-Associated Hoof Disease from free-roaming wild elk (Cervus canadensis)
Source: Anim Microbiome. 2024 Apr 22;6:20. doi: 10.1186/s42523-024-00304-9 (PMC11036743; doi:10.1186/s42523-024-00304-9)

**Supplemental Data:**

Supplementary_file_collection_metadata.xls

Supplementary_file_sample_Treponeme_matrix.xls

Supplementary_file_association_rule.xls

Supplementary_file_OTUplot.xls

Sequence data (fasta files) can be located at (-----)

| Table S1: Nested PCR primers, cycle conditions amplicon product size and positive control strains used in this study. | | | | | |
| --- | --- | --- | --- | --- | --- |
| Primer Target | Primer Sequence | | Cycle Conditions | Amplicon Size (bp) | DNA from Strain used as positive control |
|  | Forward Sequence 5’ to 3’ | Reverse Sequence 5’ to 3’ |  |  |  |
| Bacterial 16S rDNA subunit (Universal) | GAA TGC TCA TCT GAT GAC GGT AAT CGA CG | TAC CTT GTT ACG ACT T | 95°5´, (94°1´, 55°3´, 72°3´) x 25 cycles, 72°7´ | 1526 |  |
| *Treponema phagedenis* | GAA ATA CTC AAG CTT AAC TTG AGA ATT GC | CTA CGC TAC CAT ATC TCT ATA ATA TTG C | 95°5´, (95°1´, 64°1´, 72°2´) x 40 cycles, 72°10´ | 400 | *Treponema phagedenis* strain 4A (46) |
| *Treponema medium* | GAA TGC TCA TCT GAT GAC GGT AAT CGA CG | CCG GCC TTA TCT CTA AGA CCT TCT ACT AG | 95°5´, (95°1´, 68°2´, 72°2´) x 40 cycles, 72°10´ | 475 | *Treponema medium* G7201 Umemoto et al. (ATCC 700293) |
| *Treponema pedis* | GGA GAT GAG GGA ATG CRT CTT CGA TG | CAA GAG TCG TAT TGC TAC GCT GAT ATA TC | 95°5´, (95°1´, 68°2´, 72°2´) x 40 cycles, 72°10´ | 424 | *Treponema pedis* T3552B Evans et al. 2009 (DSM 18691) |
| *Fusobacterium necrophorum* | GAG AGA GCT TTG CGT CC | TGG GCG CTG AGG TTC GAC | 94°5´, (94°1´, 60°0.5´, 72°2´) x 40 cycles, 72°10´ | 600 | *Fusobacterium necrophorum* subsp. *necrophorum* VPI 2891 [2358, JCM 3718] (Flugge) Moore and Holdeman (ATCC 25286) |
| ⁰= degrees Celsius; ´ = minutes | | | | |  |

Supplemental Figure S1:

Phyloseq alpha diversity of all samples using different alpha diversity indexes including anatomical sampling location (coronary band CB, heel bulb HB, interdigital ID, or other OT (usually dewclaw)) with lesion grade severity.


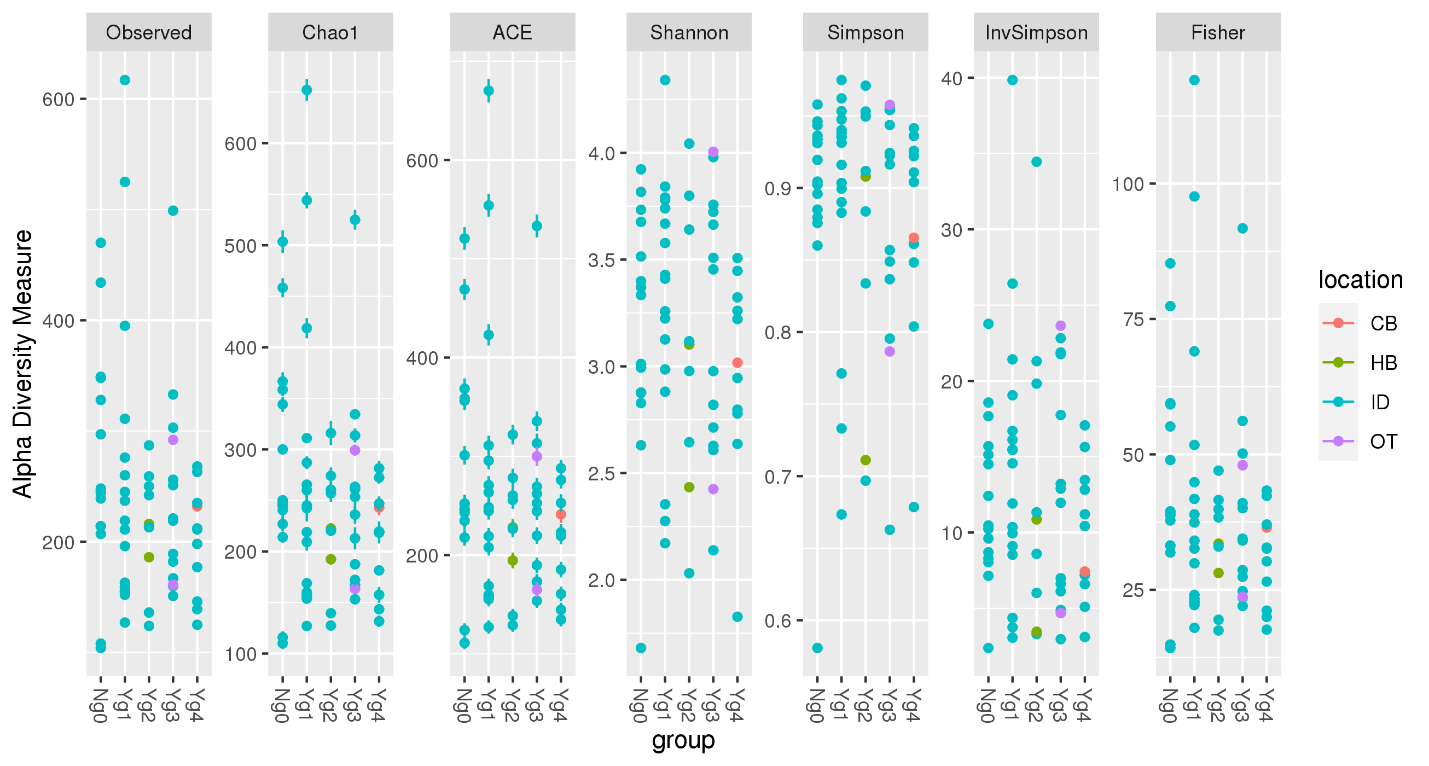


Supplemental Figure S2: Differential Heat Tree Map of Differentially expressed classified OTUs between lesion grades.

Supplemental Figure S3: OTUs significantly different in 3 out of 4 lesion grades as compared to no lesion (Ng0).


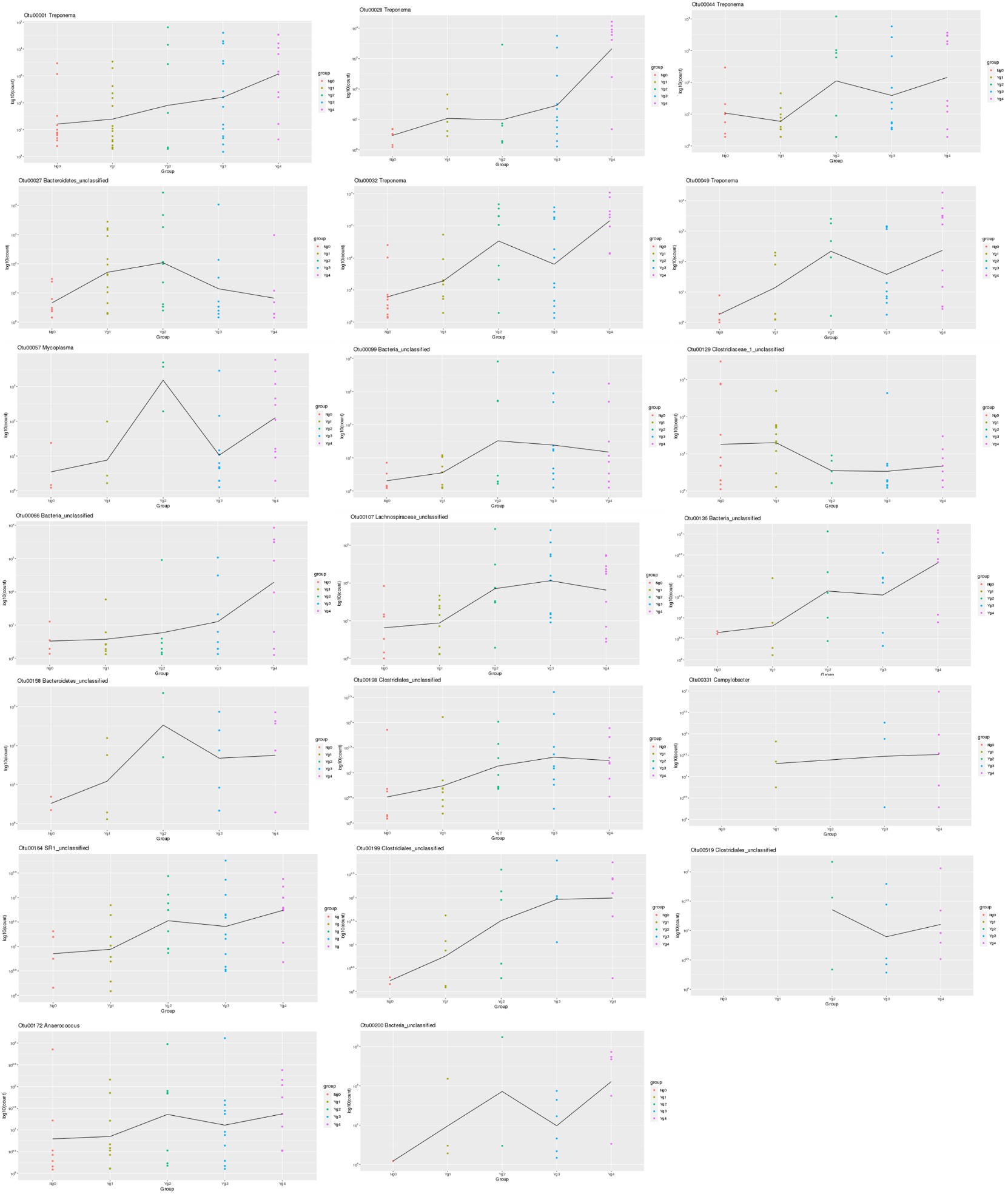


Supplemental figure S4: Significantly different OTUs in all 4 lesion grades (Yg1, Yg2, Yg3 & Yg4) as compared to no lesion (Ng0).


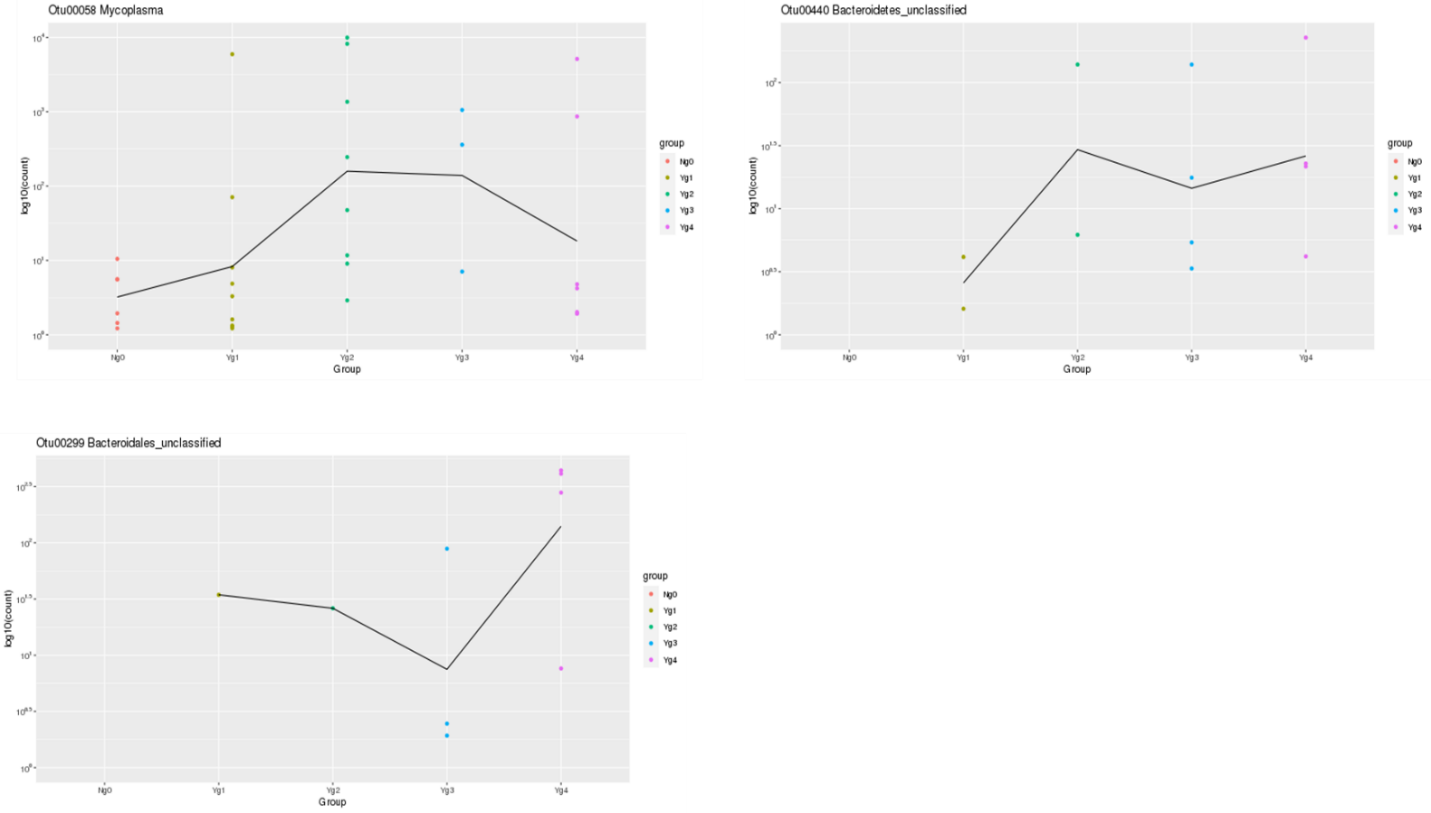

Supplement: Supplementary file 5 — Supplementary Material 5 [file 42523_2024_304_MOESM5_ESM.docx]
